# The role of biopsy in diagnosing infection after hip and knee arthroplasty: a meta-analysis

**DOI:** 10.1007/s00402-021-04323-y

**Published:** 2022-01-21

**Authors:** Cheng Li, Donara Margaryan, Carsten Perka, Andrej Trampuz

**Affiliations:** 1 Department of Orthopaedic Surgery, Beijing Jishuitan Hospital, Fourth Clinical College of Peking University, Beijing, People’s Republic of China; 2 Charité-Universitätsmedizin Berlin, corporate member of Freie Universität Berlin, Humboldt-Universität zu Berlin, and Berlin Institute of Health, Center for Musculoskeletal Surgery (CMSC), Charitéplatz 1, 10117 Berlin, Germany

**Keywords:** Arthroplasty, Prosthesis-related infections, Biopsy, Diagnosis, Histology, Tissue culture techniques

## Abstract

**Introduction:**

Early diagnosis of periprosthetic hip and knee infection still represents a major challenge, as no single test can achieve ideal results. Currently, multiple preoperative indicators were performed to diagnose periprosthetic joint infection (PJI) to confirm or exclude infection in the early stage. However, the diagnostic value of biopsy-related tests in diagnosing periprosthetic hip and knee infection remains unclear.

**Materials and methods:**

Publications in PubMed, Embase, and the Web of Science databases were searched systematically until October 2020. Inclusion and exclusion criteria were used for screening biopsy-related studies of the diagnosis of periprosthetic hip and knee infection.

**Results:**

Three biopsy-related tests were identified in 14 articles and further analyzed in the present meta-analysis. The combined method had the highest value for the area under the curve (0.9805), followed by histology (0.9425) and microbiological tests (0.9292). In the subgroup, statistical differences were identified in sensitivity and specificity for PJI diagnosis between the synovial fluid culture and biopsy culture group, as well as in the biopsy-related combined method and serum C-reactive protein.

**Conclusions:**

Biopsy culture does not appear to be advantageous compared to synovial fluid culture in the preoperative diagnosis of periprosthetic hip and knee infection. In contrast, combined biopsy microbial culture with histology analysis shows great potential in improving the preoperative diagnosis of PJI. The standard procedure of biopsy needs to be further explored. Further research is required to verify our results.

## Introduction

Hip and knee arthroplasty have become conventional surgical methods to improve the physical function and quality of life of patients with osteoarthritis or inflammatory arthritis. Nevertheless, associated postoperative complications require attention, particularly for screening infection cases, because subsequent treatment programs significantly differed between infection and non-infection [1]. Although multiple preoperative serological and synovial fluid examinations were applied in the clinical diagnosis of periprosthetic joint infection (PJI), distinguishing or excluding early stage PJI remains challenging. This is because no single preoperative test could accurately diagnose infection, and there remains a lack of reliable evidence from microbial information [2]. Synovial fluid culture is the most commonly used preoperative test that identifies microorganisms from planktonic bacteria. Its diagnostic accuracy is lower than intraoperative tests of periprosthetic tissue and sonication fluid culture from biofilms [3–6]. Furthermore, joint fluid collection is limited in the case of dry tap [7]. To provide more reliable information before revision surgery, preoperative biopsy was used in these years, contributing to microbiologic or histologic information [8–10]. A recent meta-analysis assessed the diagnostic value of biopsy in periprosthetic shoulder infection, concluding that biopsy may help diagnose PJI of the shoulder [11]. However, the role of biopsy for the preoperative diagnosis of periprosthetic hip and knee infection remains controversial [12–15]. Some reports found the biopsy-related method to not be advantageous over conventional synovial fluid culture [14, 15]. In addition, the biopsy-related method has been demonstrated to show better results compared to serological or synovial fluid tests [13, 16, 17].

The current study aimed to investigate the diagnostic value of the biopsy-related method in the diagnosis of periprosthetic hip and knee infection, and investigated whether biopsy results are superior to that of other preoperative conventional methods.

## Materials and methods

The present study followed the Preferred Reporting Items for Systematic Reviews and Meta-Analysis (PRISMA) guidelines [18].

### Search strategy and criteria

Three electronic databases (Web of Science, PubMed, and Embase) were used in this meta-analysis. The medical subject headings or keywords were referred to in previous studies [2, 11]: “arthroplasty or hemiarthroplasty or joint prosthesis or joint replacement or periprosthetic joint or prosthetic joint”, “infection or infectious or infected”, “biopsy”. The retrieval period was from the establishment of each database to October 2020.

Literature was selected in accordance with the inclusion and exclusion criteria. Inclusion criteria were: (1) human studies related to preoperative biopsy in periprosthetic hip or knee infection; (2) clear description of the definition of PJI in the manuscript; and (3) provision of the numerical values of true-positive (TP), false-positive (FP), true-negative (TN), and false-negative (FN). Exclusion criteria were: (1) animal experiments, case reports, conference papers, duplicate studies, meta-analysis, and systematic reviews; (2) biopsy site was not related to the location of hip or knee replacement; and (3) details of diagnostic information or full-text article was not available.

To compare the diagnostic accuracy between biopsy-related and other preoperative methods used in the diagnosis of PJI, diagnostic methods that occurred on more than four occasions were further analyzed in this research.

### Data acquisition and study quality assessment

The following information was extracted by two independent investigators an orthopedic surgeon and infectious disease specialist: author, antimicrobial administration, biopsy method, country, diagnostic criteria, non-microbiological test or microbiological test from the selected study, sample size, study design, surgical site, sensitivity, specificity, year of publication, and values of TP, FP, TN, and FN. Cross-checking of the results was performed by these two authors, and an expert in surgical infection as an adjudicator to determine the disagreements. Quality assessment of all identified biopsy-related studies was evaluated using the Quality Assessment of Diagnostic Accuracy Studies (QUADAS-2) guidelines [19].

### Statistical methods

To assess the diagnostic value of biopsy-related methods for PJI detection, the pooled sensitivity, specificity, positive likelihood ratio (PLR), negative likelihood ratio (NLR), as well as diagnostic odds ratio (DOR) and area under the curve (AUC) value were calculated using MetaDiSc 1.4 (Hospital Universitario Ramón y Cajal, Madrid, Spain). The level of heterogeneity was identified using the *I*^2^ statistic, with *I*^2^ values of 0–25% indicating low heterogeneity, 51–75% indicating moderate heterogeneity, and > 75% indicating high heterogeneity. A random-effects model was used in significant heterogeneity and a fixed-effects model in the case of non-significant heterogeneity. The potential source of heterogeneity was further explored. Deeks’ funnel plot was used to explore the potential for publication bias.

For further comparison, the diagnostic accuracy between biopsy-related and other conventional preoperative methods in the diagnosis of PJI, logit-transformed sensitivity, specificity, and corresponding 95% confidence interval (CI) of the index tests were compared using *z* test statistics. *P* values ≤ 0.05 were considered statistically significant.

## Results

**Fig. 1:**
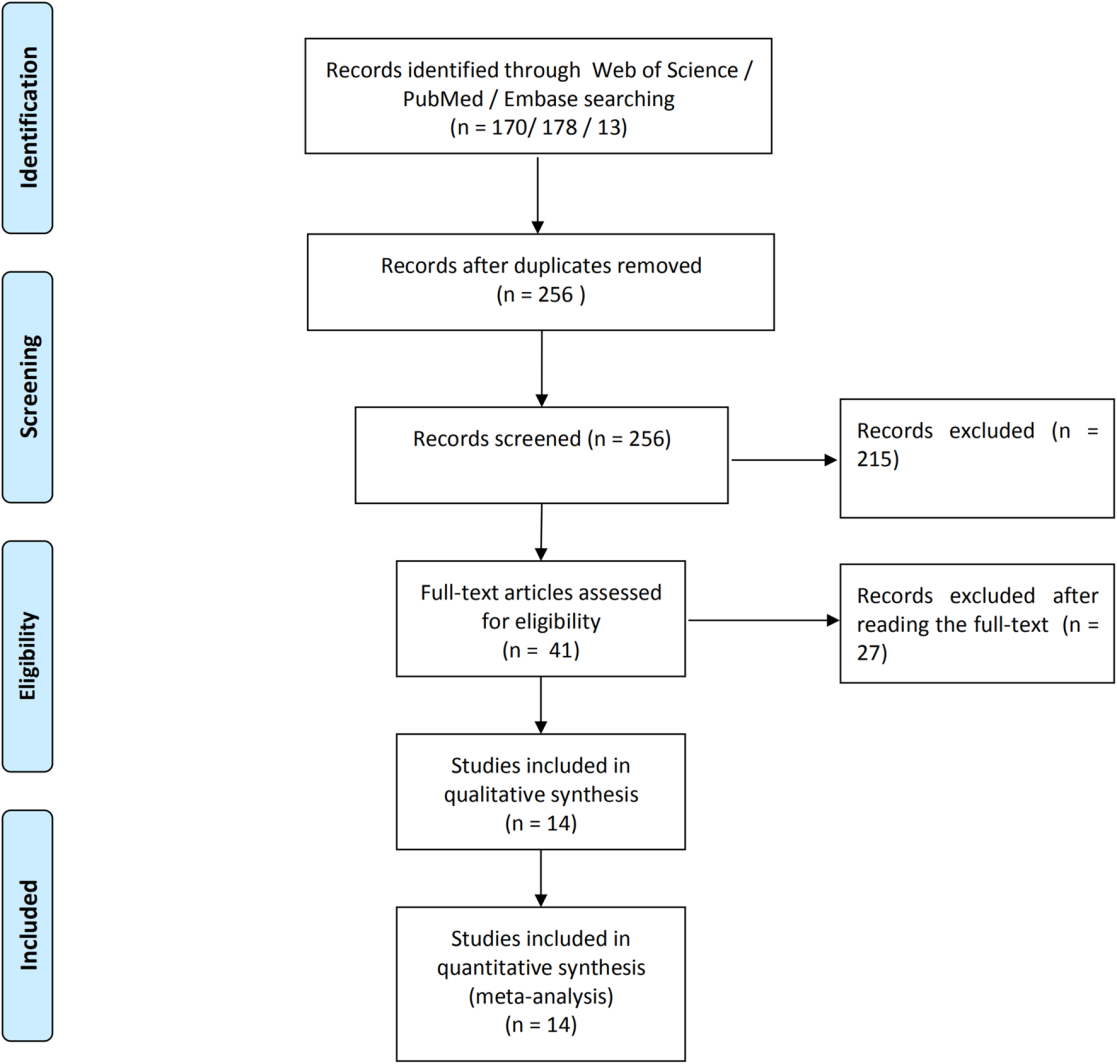
Flow diagram of the included studies in this meta-analysis

**Fig. 2:**
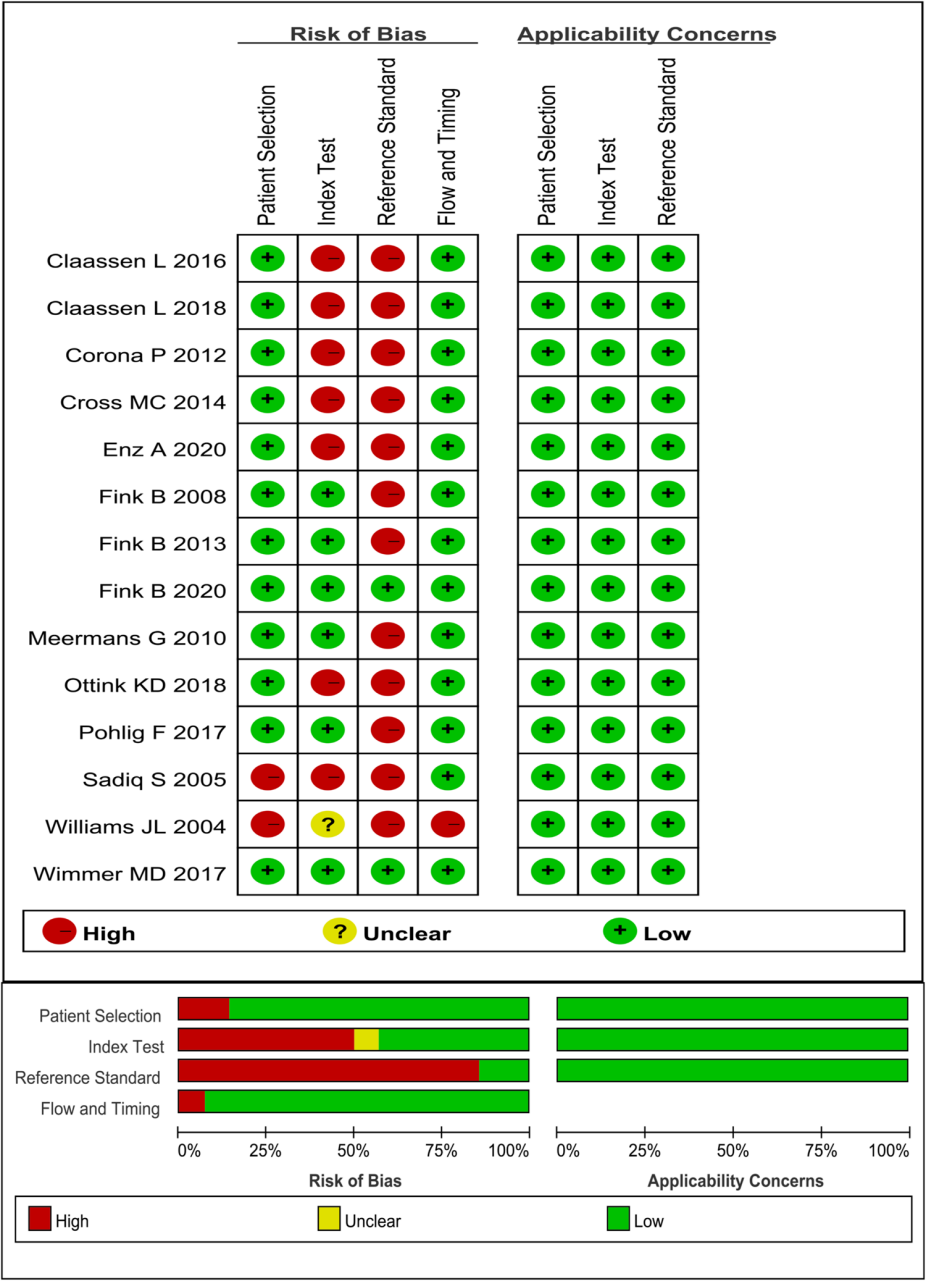
Methodological quality of the selected studies

**Fig. 3:**
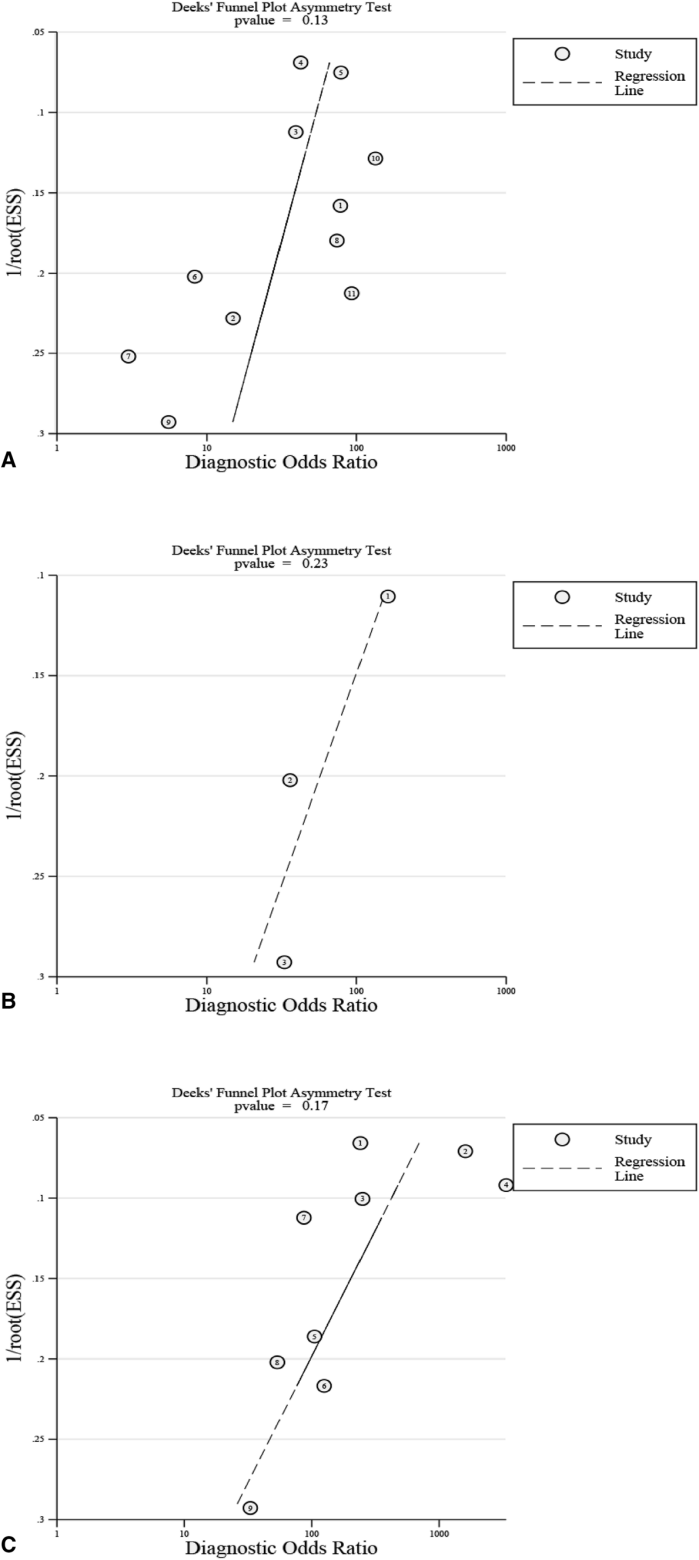
Deeks’ funnel plot to assess potential publication bias for **A** microbial culture, **B** histology, and **C** combined method

According to the inclusion and exclusion criteria, 14 articles were included in the meta-analysis (Fig. 1) [8–10, 12–17, 20–24]. Of 1698 cases, 655 were infections. The selected studies were published by five countries from 2004 to 2020, with Germany having the most number of publications (8), followed by the United Kingdom (3), and included the Netherlands, the United States and Spain, respectively (Table 1). Three biopsy-related diagnostic methods were shown in the current study, and 11 studies described microbial culture [8–10, 14, 15, 17, 21–24]. Nine studies were related to the combined method (microbial culture and histology) [8–10, 12, 13, 16, 17, 20], whereas three studies performed histopathological methods [8–10]. The QUADAS-2 quality assessments for the included studies of the meta-analysis are depicted in Fig. 2. Publication bias was evaluated by Deeks’ funnel plot analysis. There was no statistically significant findings based on this meta-analysis (Fig. 3).

**Table 1:** Characteristics of the selected studies

Reference	Year	Country	Study design	Location	Number of patients	Fluoroscopic, ultrasound, or arthroscopy guidance	Number of samples	microbiological test or microbiological test	Sensitivity (%)	Specificity (%)	Received antibiotics	Diagnostic standard
[12]	2020	Germany	Prospective	Hip	508	Fluoroscopic	5	Microbial culture + Histology	93.80	94.10	NO	MSIS
	2020	Germany	Prospective	Knee		Fluoroscopic	5	Microbial culture + Histology	93.80	99.10	NO	MSIS
[13]	2013	Germany	Prospective	Hip	100	Fluoroscopic	5	Microbial culture + Histology	82	98	NO	M, H
[16]	2008	Germany	Prospective	Knee	145	Arthroscopy guidance for histology	5	Microbial culture + Histology	100	98.10	NO	M, H
[20]	2016	Germany	Prospective	Hip	30	Fluoroscopic	≥ 2	Microbial culture + Histology	85	100	NA	MSIS
[21]	2010	UK	Prospective	Hip/ Knee	120	Fluoroscopic (hip)	≥ 3	Microbial culture	79.10	100	NO	M
[17]	2017	Germany	Prospective	Hip	20	Arthroscopy	5	Microbial culture	75	83.30	NO	M, H
	2017	Germany	Prospective	Hip		Arthroscopy	5	Microbial culture + Histology	87.50	100	NO	M, H
[8]	2020	Germany	Retrospective	Hip/ Knee	102	Fluoroscopic	1	Microbial culture	51.90	97.30	NA	M, H
	2020	Germany	Retrospective	Hip/ Knee		Fluoroscopic	1	Histology	51.90	100	NA	M, H
	2020	Germany	Retrospective	Hip/ Knee		Fluoroscopic	1	Microbial culture + Histology	70.40	97.30	NA	M, H
[15]	2004	UK	Retrospective	Hip	273	NA	NA	Microbial culture	83	90	YES	M
[22]	2005	UK	Retrospective	Hip/ Knee	159	Fluoroscopic	NA	Microbial culture	88	91	NO	M
[9]	2016	Germany	Retrospective	Knee	34	Arthroscopy	5	Microbial culture	25	96	NA	M, H
	2016	Germany	Retrospective	Knee		Arthroscopy	5	Histology	75	92	NA	M, H
	2016	Germany	Retrospective	Knee		Arthroscopy	5	Microbial culture + Histology	88	88	NA	M, H
[23]	2018	Netherlands	Retrospective	Hip	16	Ultrasound	≥ 1	Microbial culture	33	85	NO	M
	2018	Netherlands	Retrospective	Hip		Fluoroscopic	≥ 4	Microbial culture	82	100	NO	M
[10]	2018	Germany	Retrospective	Hip	10	Arthroscopy	5	Microbial culture + Histology	100	83	NA	M, H
	2018	Germany	Retrospective	Hip		Arthroscopy	5	Histology	100	83	NA	M, H
	2018	Germany	Retrospective	Hip		Arthroscopy	5	Microbial culture	25	100	NA	M, H
[14]	2014	USA	Retrospective	Hip	110	Fluoroscopic	NA	Microbial culture	41	100	Yes	M
[24]	2012	Spain	Retrospective	Hip/ Knee	24	Fluoroscopic	≥ 2	Microbial culture	88.20	100	NO	M, H, P

H = histological examination, MSIS = Musculoskeletal Infection Society, M = microbiological or laboratory examination, NA = not available, P = presence of sinus tract or purulence around the prosthesis

### Diagnostic accuracy of three different biopsy-related tests

**Fig. 4:**
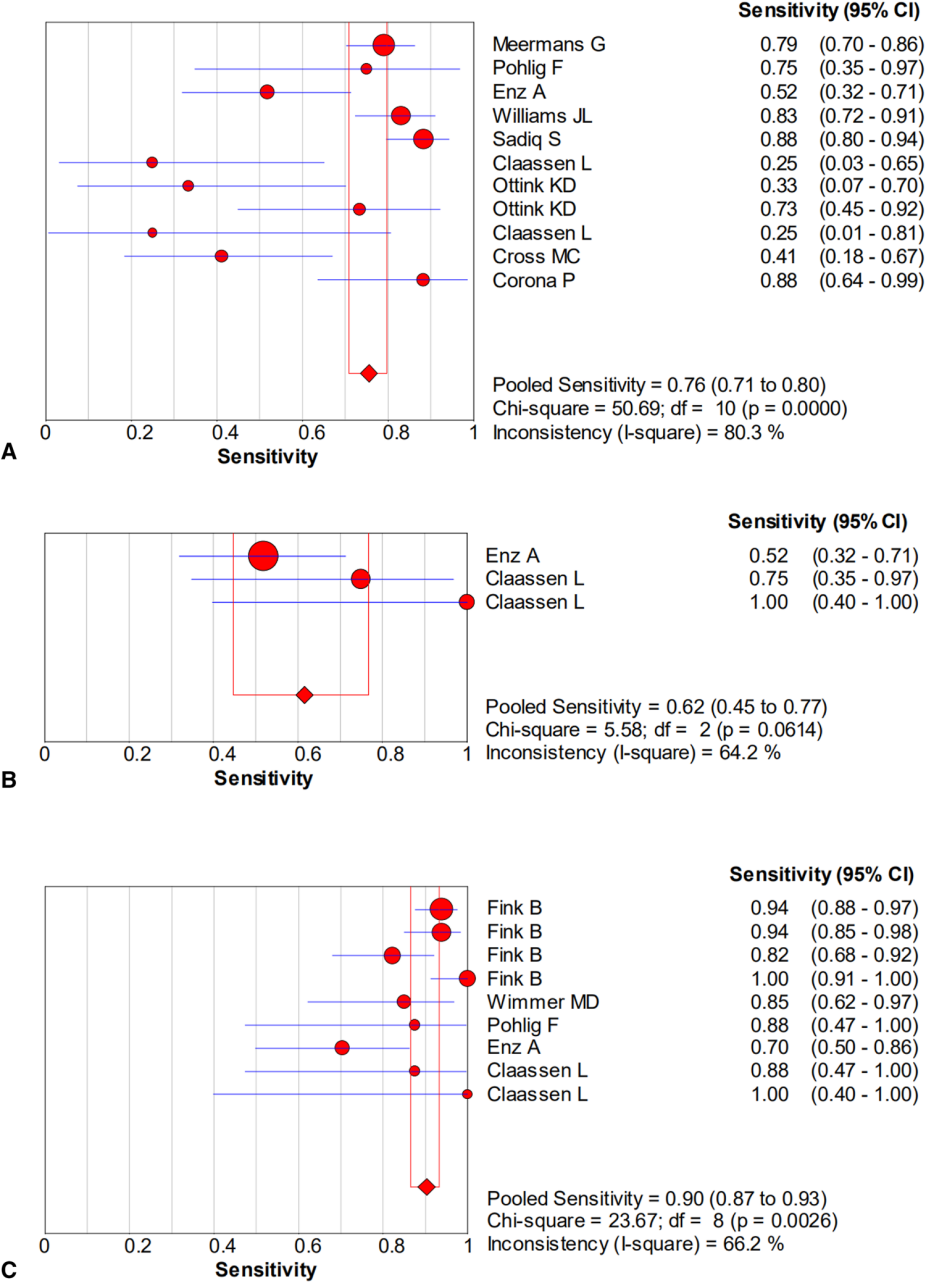
Forest plots of sensitivity for the biopsy of **A** microbial culture, **B** histological analysis, and **C** the combined method

**Fig. 5:**
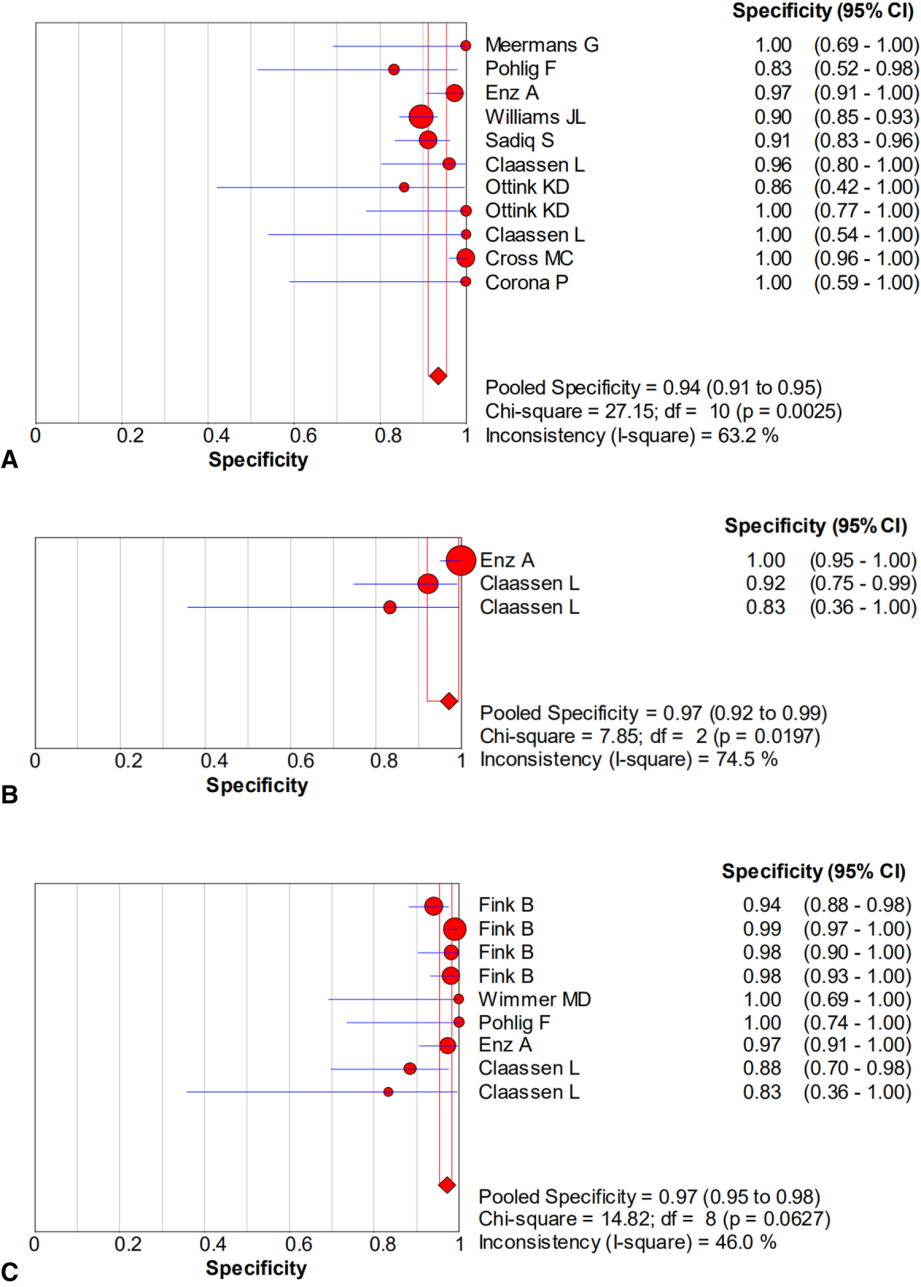
Forest plots of specificity for the biopsy of **A** microbial culture, **B** histological analysis, and **C** the combined method

**Fig. 6:**
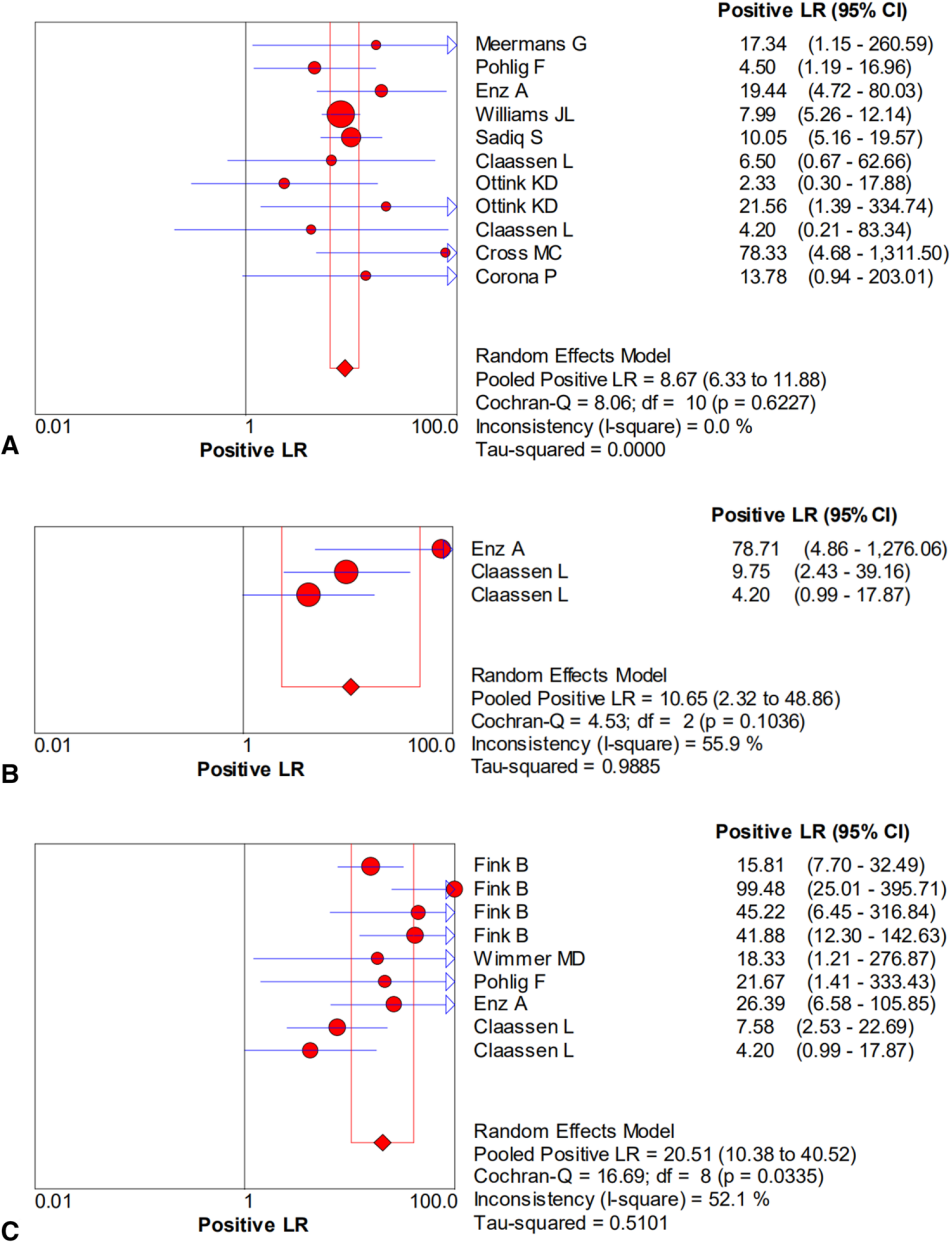
Forest plots of the positive likelihood ratio for the biopsy of **A** microbial culture, **B** histological analysis, and **C** the combined method

**Fig. 7:**
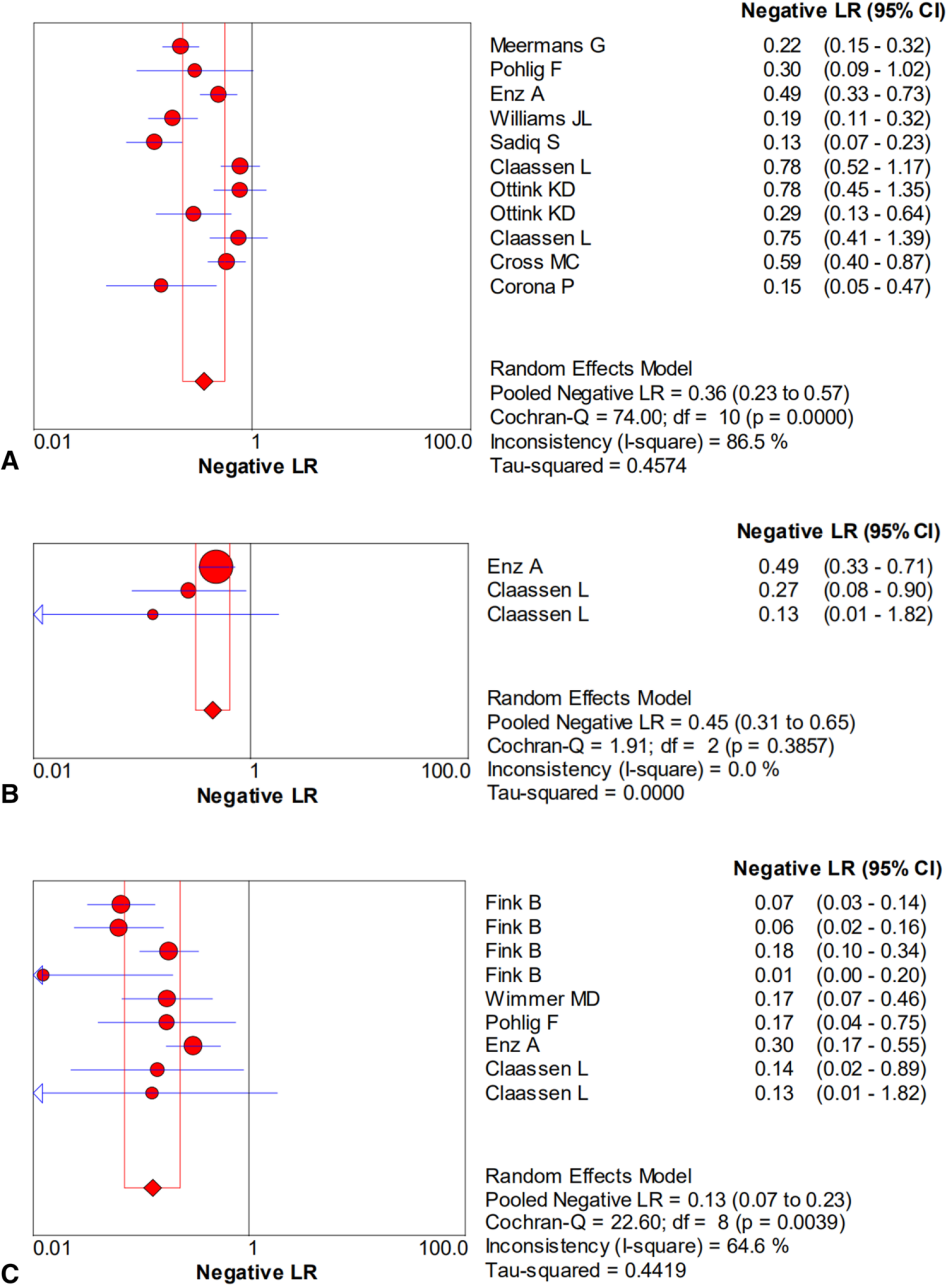
Forest plots of the negative likelihood ratio for the biopsy of **A** microbial culture, **B** histological analysis, and **C** the combined method

**Fig. 8:**
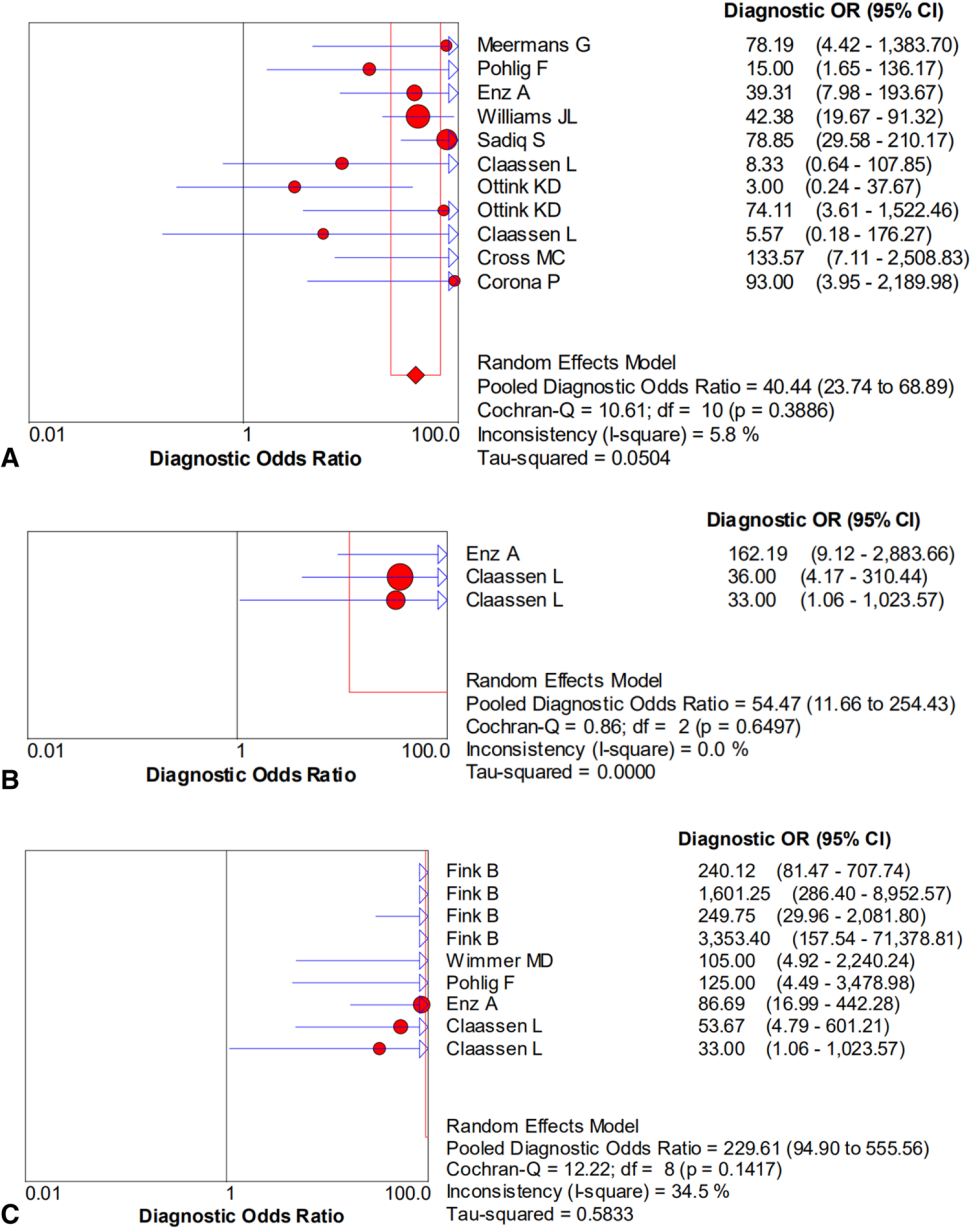
Forest plots of the diagnostic odds ratio for the biopsy of **A** microbial culture, **B** histological analysis, and **C** the combined method

**Fig. 9:**
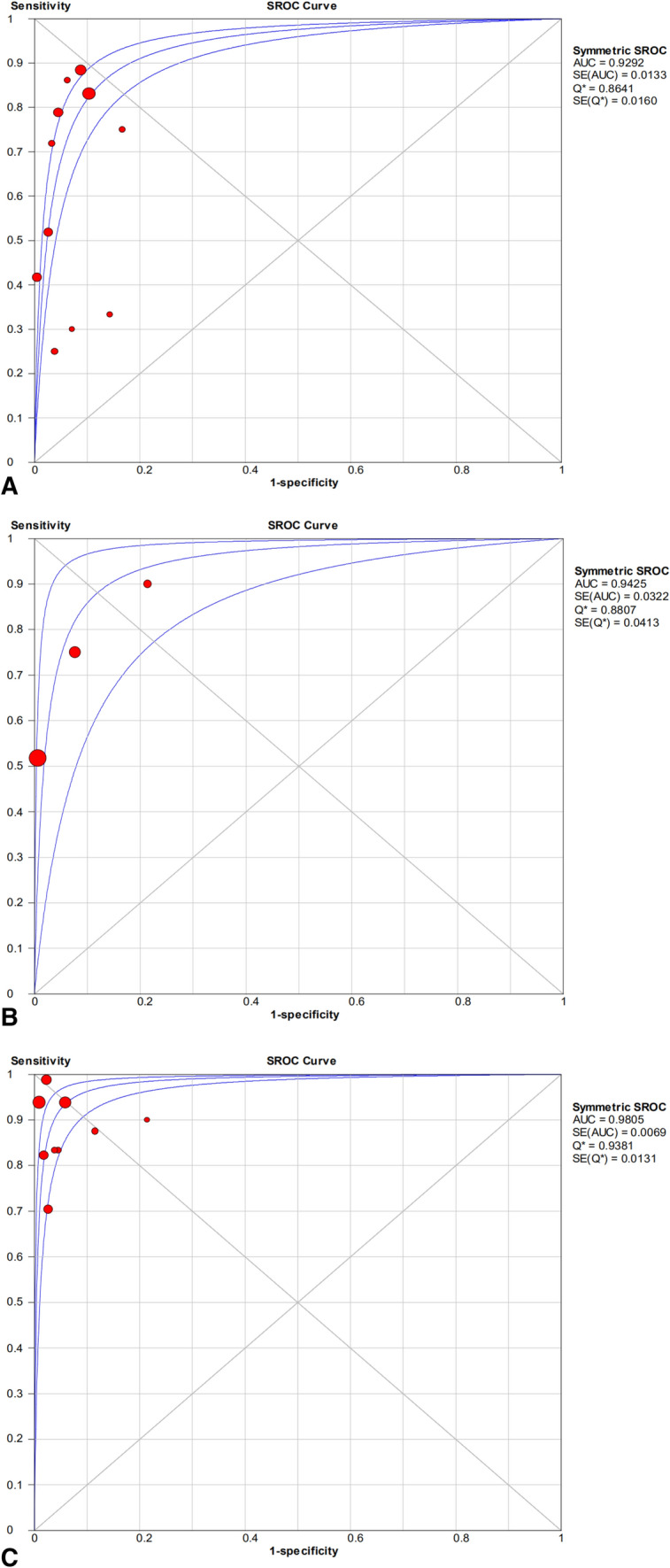
Summary of SROC for the biopsy of **A** microbial culture, **B** histological analysis, and **C** the combined method

The pooled sensitivity, specificity, PLR, NLR, and DOR estimates for the diagnosis of PJI using microbiological tests were 0.76 (95% CI 0.71–0.80), 0.94 (95% CI 0.91–0.95), 8.67 (95% CI 6.33–11.88), 0.36 (95% CI 0.23–0.57), and 40.44 (95% CI 23.74–68.89), respectively (Figs. 4A, 5A, 6A, 7A, 8A). The summary receiver operating characteristic (SROC) plot showed the sensitivity, specificity, and 95% confidence and prediction regions, with an area under the curve (AUC) of 0.9292 (standard error of 0.0133; Fig. 9A).

The overall pooled sensitivity, specificity, PLR, NLR, and DOR of the histology for PJI were 0.62 (95% CI 0.45–0.77), 0.97 (95% CI 0.92–0.99), 10.65 (95% CI 2.32–48.86), 0.45 (95% CI 0.31–0.65), and 54.47 (95% CI 11.66–254.43), respectively (Figs. 4B, 5B, 6B, 7B, 8B). The AUC value was 0.9425 (standard error of 0.0322; Fig. 9B).

The overall pooled sensitivity, specificity, PLR, NLR, and DOR of the combined method for PJI were 0.90 (95% CI 0.87–0.93), 0.97 (95% CI 0.95–0.98), 20.51 (95% CI 10.38–40.52), 0.13 (95% CI 0.07–0.23), and 229.61 (95% CI 94.90–555.56), respectively (Figs. 4C, 5C, 6C, 7C, 8C). The AUC value was 0.9805 (standard error of 0.0069; Fig. 9C). Our studies showed heterogeneity, with the random-effects model used.

### Subgroup analysis

According to the predefined inclusion criteria, two conventional methods were included in the subgroup analysis. The first group compared between synovial fluid and biopsy culture, with five publications identified [14, 15, 17, 21, 23]. The sensitivity and specificity of synovial fluid culture were 0.78 (95% CI 0.72–0.84) and 0.96 (95% CI 0.93–0.98), respectively. The sensitivity and specificity of biopsy culture were 0.75 (95% CI 0.69–0.81) and 0.93 (95% CI 0.90–0.95), respectively. Both the sensitivity and specificity of synovial fluid culture were higher than that of biopsy culture. The second group compared serum C-reactive protein (CRP) and biopsy-related combined methods, with five studies [9, 10, 13, 16, 17]. The sensitivity and specificity of CRP were 0.68 (95% CI 0.58–0.76) and 0.77 (95% CI 0.71–0.83), respectively. The sensitivity and specificity of the combined method were 0.90 (95% CI 0.83–0.95) and 0.97 (95% CI 0.93–0.99), respectively. The sensitivity and specificity of the combined method were higher than those of CRP. The sensitivity and specificity of these two groups were found to be statistically significant (*P* < 0.001).

## Discussion

The current meta-analysis showed the diagnostic accuracy of three biopsy-related methods. The AUC value of the combined method was superior to both histologic and microbiologic assays in the diagnosis of periprosthetic hip and knee infection. This study also compared the diagnostic value between biopsy-related and conventional methods in diagnosing PJI, with synovial fluid culture demonstrating better results than biopsy culture (*P* < 0.001). Furthermore, the biopsy-related combined method was found to show higher sensitivity and specificity than serum CRP (*P* < 0.001).

Infection after joint arthroplasty is a topic that has received increasing attention in recent years, particularly periprosthetic hip and knee infection [25]. Previous reports found infection to be the main reason for failure in knee arthroplasty; however, in hip replacement failure, it was reported to be the third most common cause [26–28]. This complication is accompanied by high mortality rates, which are even higher than some common cancer types [29]. However, the diagnosis of PJI currently remains a major challenge. Although various tests have been performed in the diagnosis of PJI, an ideal diagnostic method that fulfills the conditions of high diagnostic accuracy, early differential diagnosis, as well as identification of pathogenic bacteria from infection cases has not yet been found. Early microbiological tests could provide the reference value for the following intraoperative diagnosis. Synovial fluid puncture and biopsy are two preoperative invasive methods that could provide information on the causative microbial agent in the diagnosis of PJI. Synovial fluid culture is the most commonly used method and recommended by some infection societies [5, 30, 31]. However, the role of biopsy in the diagnosis of PJI remained unclear. A number of studies did not recommend the routine application of biopsy, as it offers no advantage over traditional synovial fluid culture in detecting microorganisms [14, 15]. In our current study, both the sensitivity and specificity of biopsy culture were lower than that of synovial fluid culture (78% and 96% vs*.* 75% and 93%). Williams et al*.* [15] reported that the synovial fluid culture had a higher diagnostic accuracy than biopsy culture (90.1% vs*.* 87.9%), with more false-positive results in biopsy culture than synovial fluid culture (21 vs*.* 13). However, the limitation of the previously mentioned study was the culture time of specimens, which was only up to 7 days, and histology analysis was not applied to further confirm infection. Conversely, the study of Pohlig and co-workers [17] showed the diagnostic accuracy of biopsy to be greater than that of joint fluid (80% vs*.* 75%), with the 10 days of inoculation applied for all samples and histology used to assess infection. Cross and colleagues [14] found that the diagnostic accuracy of aspiration culture was superior to that of biopsy (94% vs*.* 91%). Interestingly, combining these two methods did not improve culture results. In the previously mentioned study, the authors only used intraoperative tissue culture as the gold standard, and some patients were still under antibiotic therapy before sample collection. These factors most likely affected the final results. A study by Meermans et al*.* [21] also demonstrated synovial fluid culture to yield better results than biopsy; however, the combined method showed a diagnostic accuracy of 90.8%, superior to that of single biopsy or synovial fluid culture (80.8% and 84.1%, respectively). Compared to the study by Cross and colleagues, the author discontinued antibiotic treatment in patients 4 weeks before sample collection [14, 21].

Histologic analysis is an additional biopsy-related method included in our screening, with a sensitivity of 62% and specificity of 97%. Although histologic examination did not obtain microbiologic information, it was found to have better diagnostic accuracy than biopsy culture in the diagnosis of PJI. The study of Claassen and co-workers showed the diagnostic accuracy of biopsy histology to be superior to that of biopsy culture, serum white cell count, and CRP in periprosthetic knee infection (88%, 79%, 67%, and 65%, respectively) [9]. Similar results were also reported in periprosthetic hip infection by Claassen and colleagues [10], with biopsy histology demonstrating the highest accuracy compared to biopsy culture, serum white cell count, and CRP (90%,70%, 70%, and 80%, respectively). In a recent study, Enz et al*. *[8] evaluated the use of biopsy in the diagnosis of periprosthetic hip and knee infection. Biopsy culture had a sensitivity of 51.9% and a specificity of 97%, with biopsy histology demonstrating a similar sensitivity, although higher specificity (100%). However, the combination of the two methods resulted in increased sensitivity to 70.4%. The combined method shows better results than a single biopsy of histopathological analysis or microbiological examination. Interestingly, similar results were also observed in our meta-analysis, with the combined method demonstrate a superior AUC value compared to each method alone. Some reports also reported the combined method to have a better diagnostic value than other conventional preoperative tests [9, 10, 16, 17]. A study by Pohlig et al*.* [17] found that the combined method of biopsy not only had a higher diagnostic accuracy than conventional synovial fluid culture, synovial fluid cell count/percentage neutrophils, erythrocyte sedimentation rate (ESR) and CRP (95%, 75%, 70%, 83%, and 70%, respectively), but was also superior to that of the combined method of synovial fluid cell count/percentage neutrophils plus ESR or CRP and all synovial fluid tests plus blood tests (86%, 80%, and 90%, respectively). In the subgroup analysis in our study, the diagnostic value of the combined method was greater than the conventional test using CRP in diagnosing PJI.

There was heterogeneity among the meta-analysis of this study; however, some potential factors liked affected our results. First, as no single test could achieve 100% accuracy in diagnosing PJI, the definition of PJI was used to improve the diagnostic accuracy. The diagnostic method of tissue culture, synovial fluid or sonication fluid culture, and histological examination has been recommended by some infection societies and used as one of the criteria of PJI [5, 30, 31]. However, the diagnostic approach was not uniform in our selected study, with some studies not performing histology analysis and only one study including the sonication method for culture [14, 15, 21–23]. Hence, such circumstances likely impacted the evaluation of our pooled results. Second, joint biopsy was performed by ultrasound, fluoroscopic, or arthroscopy guidance. It remains unclear whether differences among these three methods in sample collection exist. Only the study by Ottink et al. [23] reported biopsy under ultrasound and fluoroscopic guidance for the diagnosis of PJI. Here, the fluoroscopic-guided group was found to have better sensitivity and specificity than the ultrasound-guided group (82% and 100%, 33% and 85%, respectively). Third, the anatomical sites in the hip and knee differ, with biopsy sample collection of the knee easier than that of the hip to achieve the suspected site of biofilm colonization. This factor is most likely responsible for the more optimal biopsy results observed from the knee than the hip [12]. Fourth, the standard procedure of biopsy is still required in further studies. Based on the recommended clinical practice guidelines by the Infectious Diseases Society of America in the diagnosis of PJI, the optimal number of tissue samples for microbiological diagnosis is five to six, with a prolonged incubation of up to 14 days [31]. However, some the included studies did not meet these standards or presented unclear information [8–10, 14, 15, 17, 20, 22, 23]. Different instruments were used for sample collection across the various studies; however, it remains unknown which instrument is safer and obtains more reliable samples from the surgical site. In addition, using the different culture media for culture also affects the diagnostic accuracy. Previous reports found that for periprosthetic tissue specimens, the use of blood culture bottles had a better sensitivity and specificity than conventional medium in the diagnosis of PJI [32].

## Conclusions

A preoperative biopsy can be useful for diagnosing periprosthetic hip and knee infection. The combination of biopsy microbial culture and histology was found to have a higher diagnostic value than their individual use and was superior to the conventional CRP test. However, biopsy culture does not appear to hold any advantage over synovial fluid culture. Due to the lack of a unified, standardized biopsy procedure, further studies are still required to further improve the procedure and verify our results.

## Abbreviations

AUC: Area under the curve
C: Clinical signs of infection
CI: Confidence interval
CRP: C-reactive protein
DOR: Diagnostic odds ratio
ESR: Erythrocyte sedimentation rate
FN: False-negative
FP: False-positive
H: Histological examination
M: Microbiological or laboratory examination
MSIS: Musculoskeletal Infection Society
NLR: Negative likelihood ratio
NA: Not available
P: Presence of sinus tract or purulence around the prosthesis
PJI: Periprosthetic joint infection
PLR: Positive likelihood ratio
QUADAS-2: Quality Assessment of Diagnostic Accuracy Studies-2
SROC: Summary receiver operating characteristic
TN: True negative
TP: True positive

## References

[1] Li C, Renz N, Trampuz A, Ojeda-Thies C (2020). Twenty common errors in the diagnosis and treatment of periprosthetic joint infection. Int Orthop.

[2] Li C, Ojeda-Thies C, Xu C, Trampuz A (2020). Meta-analysis in periprosthetic joint infection: a global bibliometric analysis. J Orthop Surg Res.

[3] Schulz P, Dlaska CE, Perka C (2020). Preoperative synovial fluid culture poorly predicts the pathogen causing periprosthetic joint infection. Infection.

[4] Rothenberg AC, Wilson AE, Hayes JP (2017). Sonication of arthroplasty implants improves accuracy of periprosthetic joint infection cultures. Clin Orthop Relat Res.

[5] Li C, Renz N, Trampuz A (2018). Management of periprosthetic joint infection. Hip Pelvis.

[6] Fernández-Sampedro M, Fariñas-Alvarez C, Garces-Zarzalejo C (2017). Accuracy of different diagnostic tests for early, delayed and late prosthetic joint infection. BMC Infect Dis.

[7] Deirmengian C, Feeley S, Kazarian GS, Kardos K (2020). Synovial fluid aspirates diluted with saline or blood reduce the sensitivity of traditional and contemporary synovial fluid biomarkers. Clin Orthop Relat Res.

[8] Enz A, Becker J, Warnke P (2020). Increased diagnostic certainty of periprosthetic joint infections by combining microbiological results with histopathological samples gained via a minimally invasive punching technique. J Clin Med Res.

[9] Claassen L, Ettinger S, Pastor M-F (2016). The value of arthroscopic neosynovium biopsies to diagnose periprosthetic knee joint low-grade infection. Arch Orthop Trauma Surg.

[10] Claassen L, Wirries N, Ettinger S (2018). Diagnosing periprosthetic hip joint low-grade infection via arthroscopic neo synovium biopsies. Technol Health Care.

[11] Cotter EJ, Winzenried AE, Polania-Gonzalez E (2020). Role of pre-revision tissue biopsy in evaluation of painful shoulder arthroplasty: a systematic review & meta-analysis. J Shoulder Elbow Surg.

[12] Fink B, Schuster P, Braun R (2020). The diagnostic value of routine preliminary biopsy in diagnosing late prosthetic joint infection after hip and knee arthroplasty. Bone Joint J.

[13] Fink B, Gebhard A, Fuerst M (2013). High diagnostic value of synovial biopsy in periprosthetic joint infection of the hip. Clin Orthop Relat Res.

[14] Cross MC, Kransdorf MJ, Chivers FS (2014). Utility of percutaneous joint aspiration and synovial biopsy in identifying culture-positive infected hip arthroplasty. Skeletal Radiol.

[15] Williams JL, Norman P, Stockley I (2004). The value of hip aspiration versus tissue biopsy in diagnosing infection before exchange hip arthroplasty surgery. J Arthroplasty.

[16] Fink B, Makowiak C, Fuerst M (2008). The value of synovial biopsy, joint aspiration and C-reactive protein in the diagnosis of late peri-prosthetic infection of total knee replacements. J Bone Joint Surg Br.

[17] Pohlig F, Mühlhofer HML, Lenze U (2017). Diagnostic accuracy of arthroscopic biopsy in periprosthetic infections of the hip. Eur J Med Res.

[18] Moher D, Shamseer L, Clarke M (2015). Preferred reporting items for systematic review and meta-analysis protocols (PRISMA-P) 2015 statement. Syst Rev.

[19] Whiting PF, Rutjes AWS, Westwood ME (2011). QUADAS-2: a revised tool for the quality assessment of diagnostic accuracy studies. Ann Intern Med.

[20] Wimmer MD, Ploeger MM, Friedrich MJ (2017). Pre-operative intra-articular deep tissue sampling with novel retrograde forceps improves the diagnostics in periprosthetic joint infection. Int Orthop.

[21] Meermans G, Haddad FS (2010). Is there a role for tissue biopsy in the diagnosis of periprosthetic infection?. Clin Orthop Relat Res.

[22] Sadiq S, Wootton JR, Morris CA, Northmore-Ball MD (2005). Application of core biopsy in revision arthroplasty for deep infection. J Arthroplasty.

[23] Ottink KD, Wouthuyzen-Bakker M, Kampinga GA (2018). Puncture protocol in the diagnostic work-up of a suspected chronic prosthetic joint infection of the hip. J Arthroplasty.

[24] Corona P, Gil E, Guerra E (2012). Percutaneous interface biopsy in dry-aspiration cases of chronic periprosthetic joint infections: a technique for preoperative isolation of the infecting organism. Int Orthop.

[25] Li C, Ojeda-Thies C, Renz N (2020). The global state of clinical research and trends in periprosthetic joint infection: a bibliometric analysis. Int J Infect Dis.

[26] Dobzyniak M, Fehring TK, Odum S (2006). Early failure in total hip arthroplasty. Clin Orthop Relat Res.

[27] Lum ZC, Shieh AK, Dorr LD (2018). Why total knees fail—a modern perspective review. World J Orthop.

[28] Koh CK, Zeng I, Ravi S (2017). Periprosthetic joint infection is the main cause of failure for modern knee arthroplasty: an analysis of 11,134 knees. Clin Orthop Relat Res.

[29] Zmistowski B, Karam JA, Durinka JB (2013). Periprosthetic joint infection increases the risk of one-year mortality. J Bone Joint Surg Am.

[30] Parvizi J, Tan TL, Goswami K (2018). The 2018 definition of periprosthetic hip and knee infection: an evidence-based and validated criteria. J Arthroplasty.

[31] Osmon DR, Berbari EF, Berendt AR (2013). Diagnosis and management of prosthetic joint infection: clinical practice guidelines by the Infectious Diseases Society of America. Clin Infect Dis.

[32] Hughes HC, Newnham R, Athanasou N (2011). Microbiological diagnosis of prosthetic joint infections: a prospective evaluation of four bacterial culture media in the routine laboratory. Clin Microbiol Infect.

